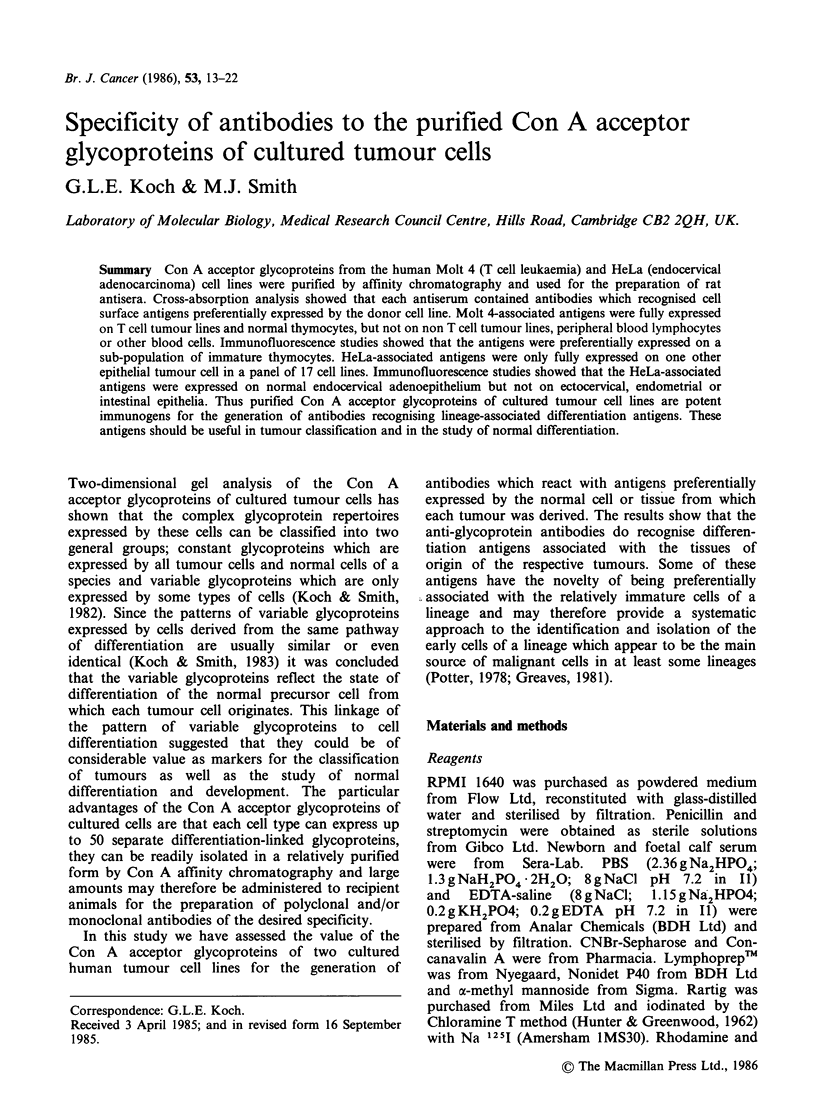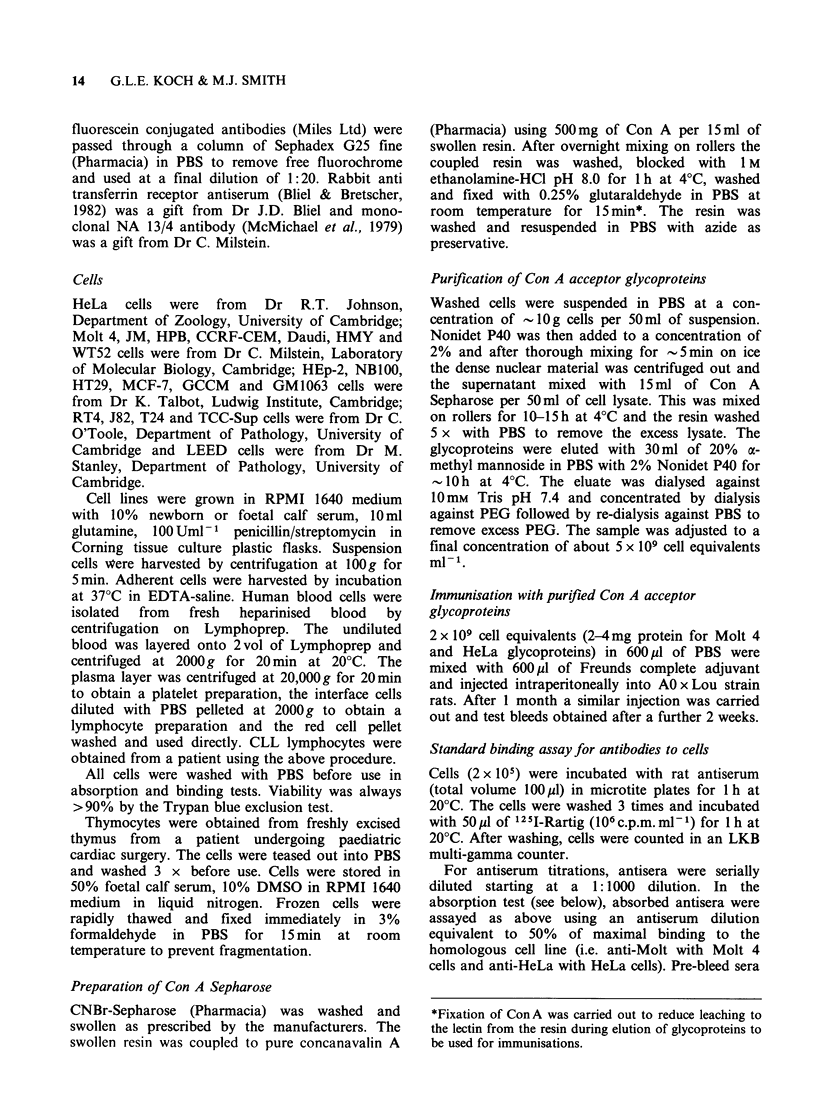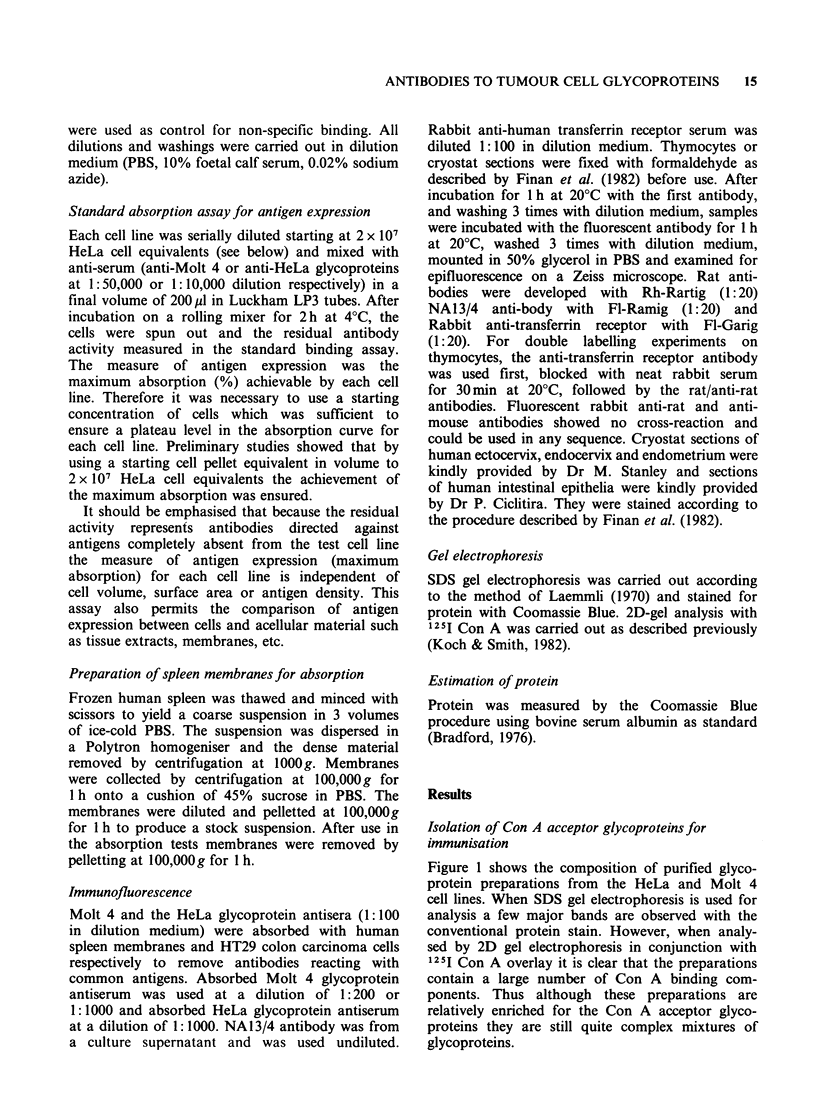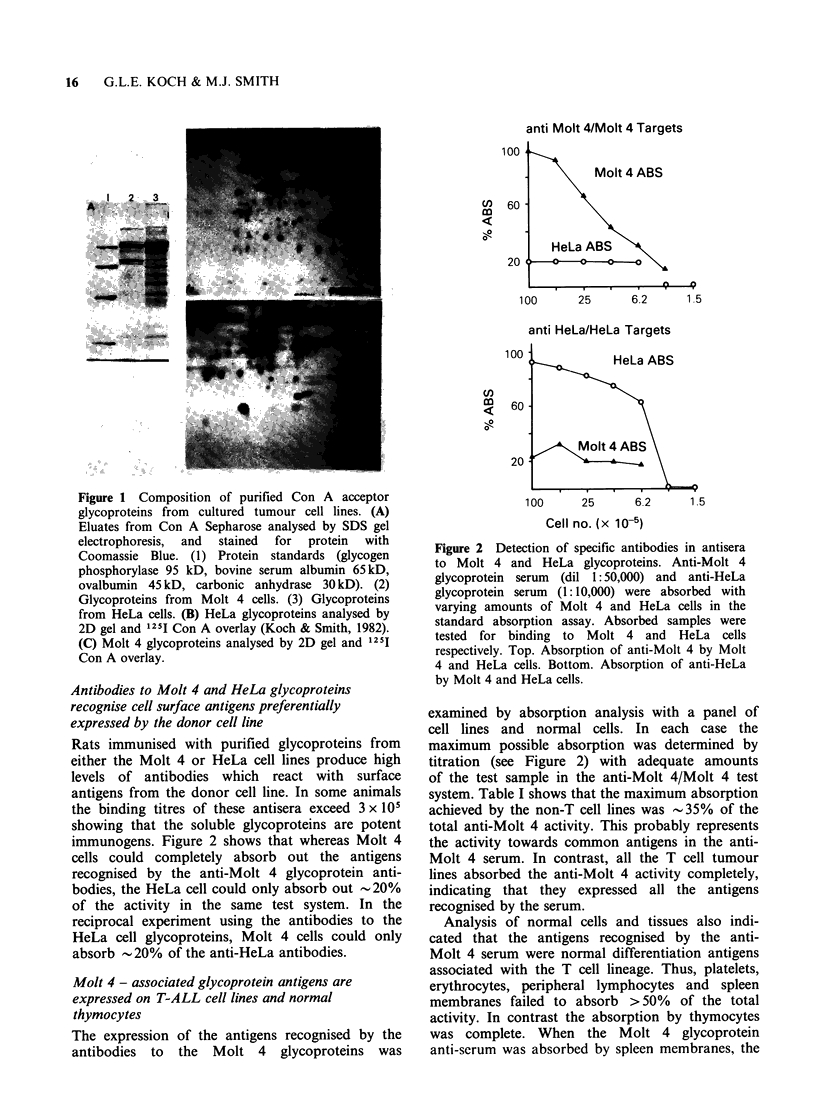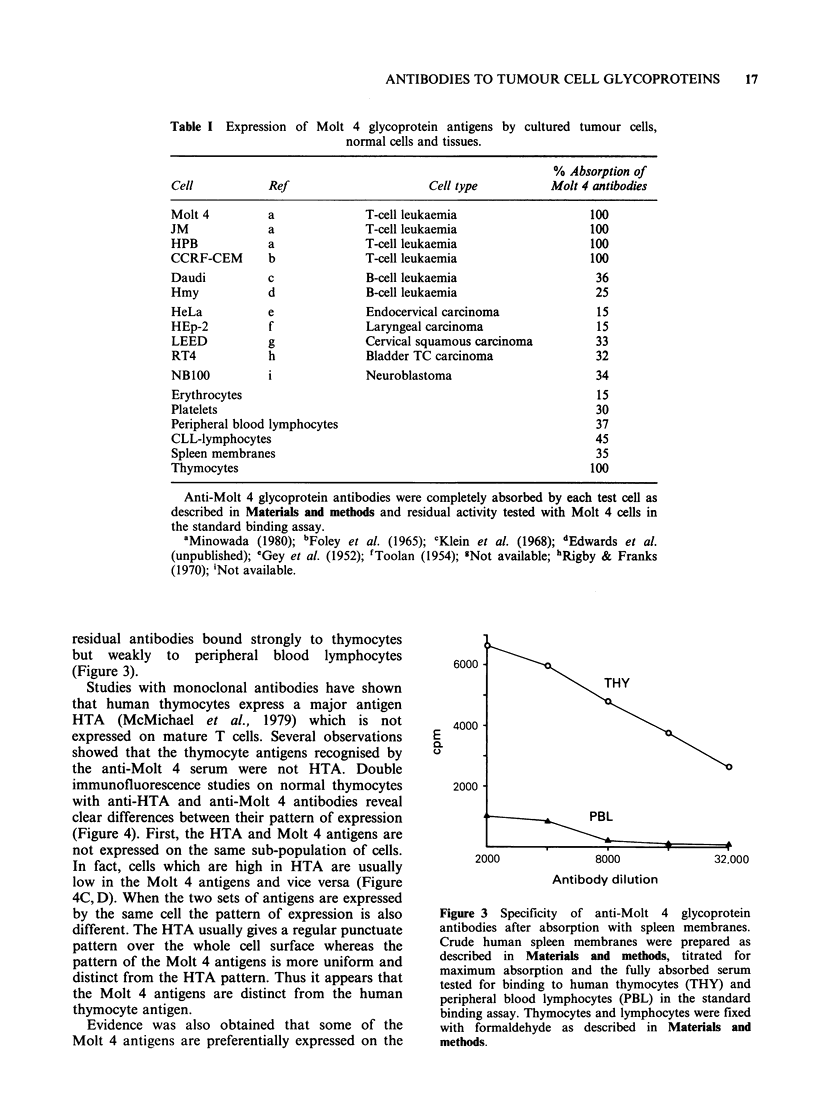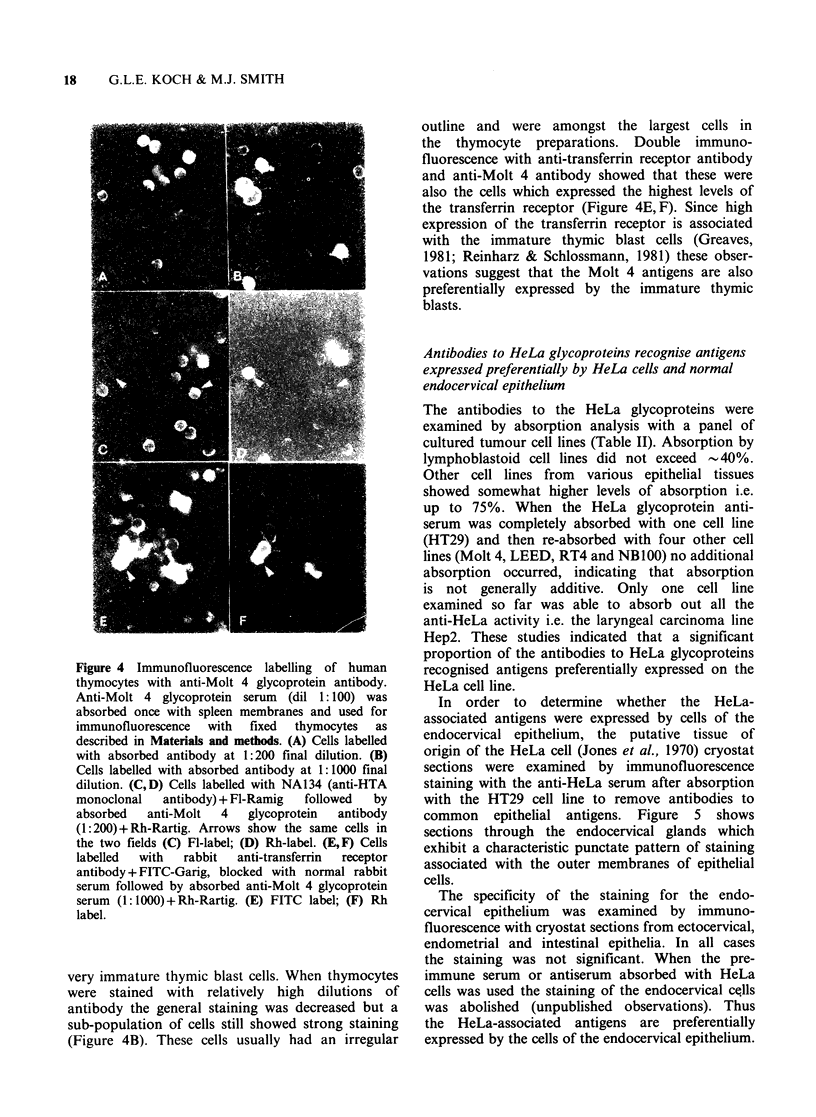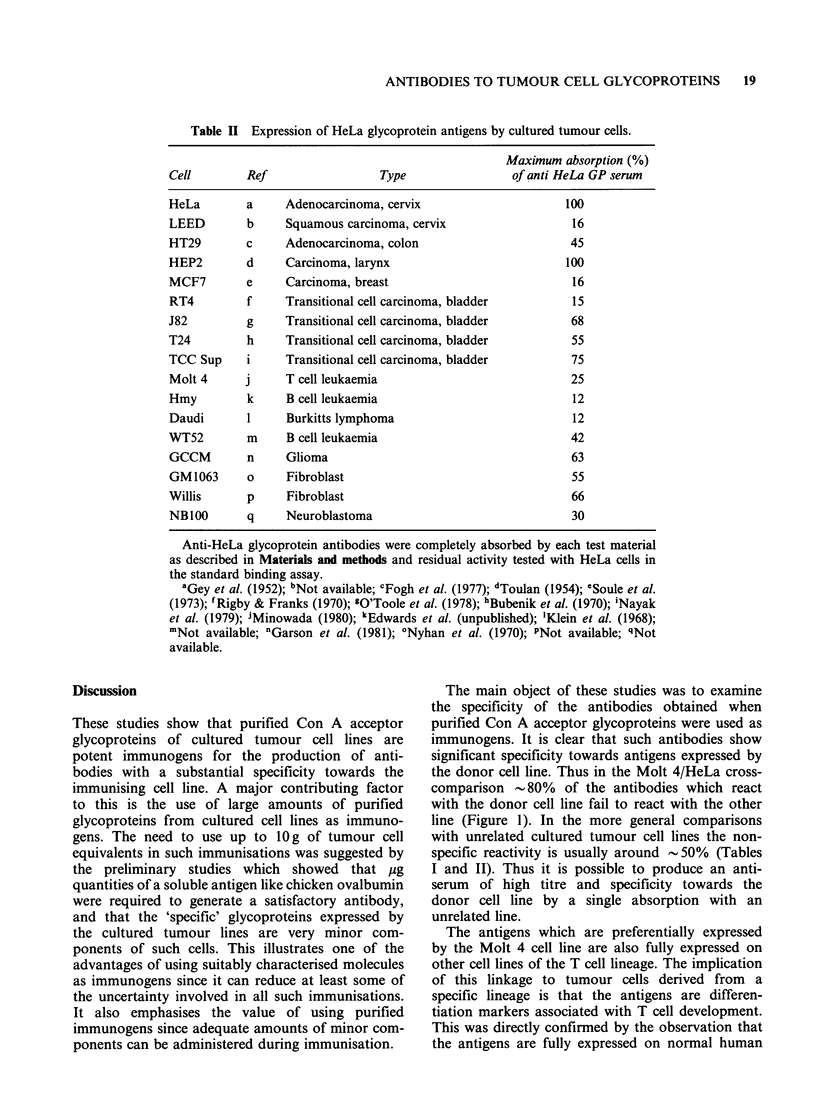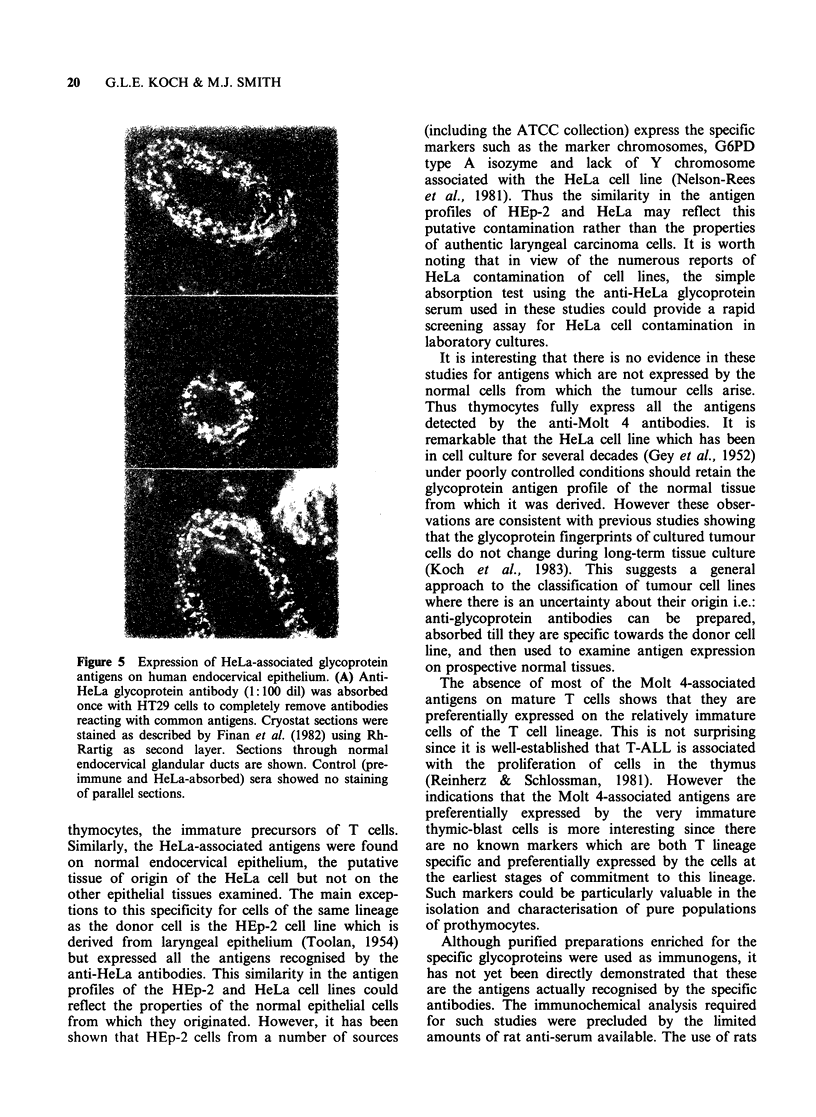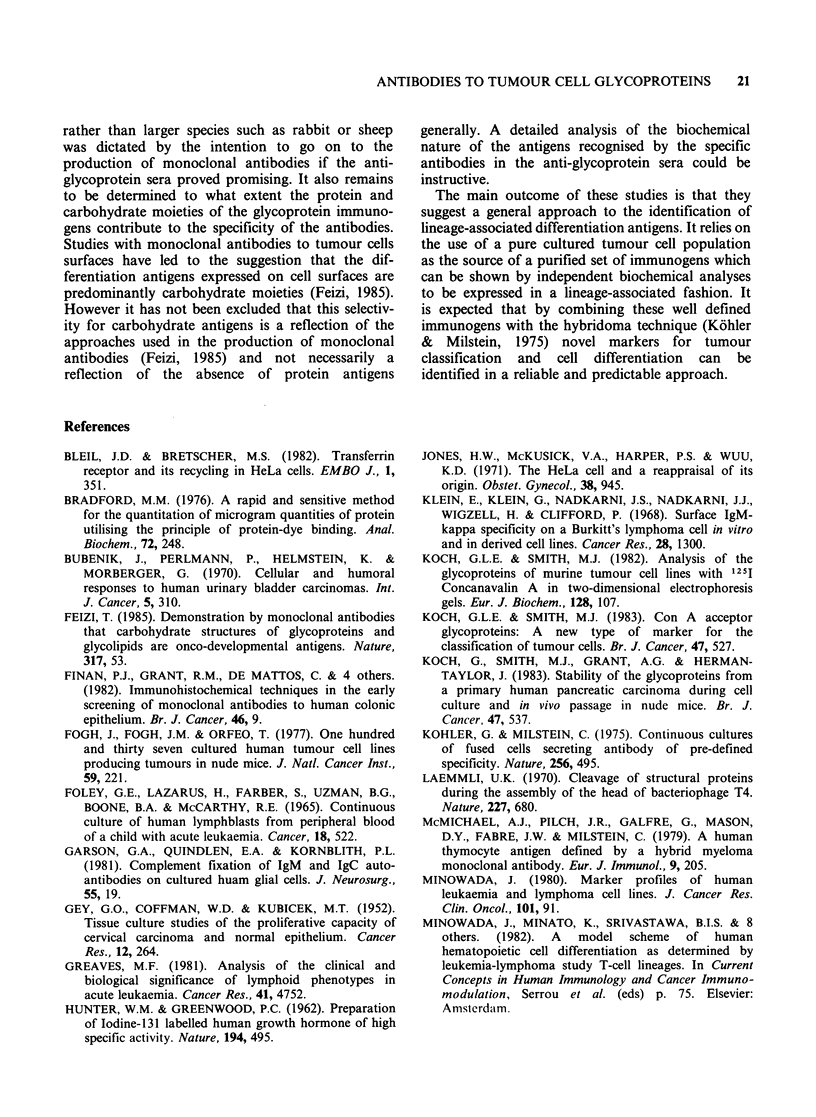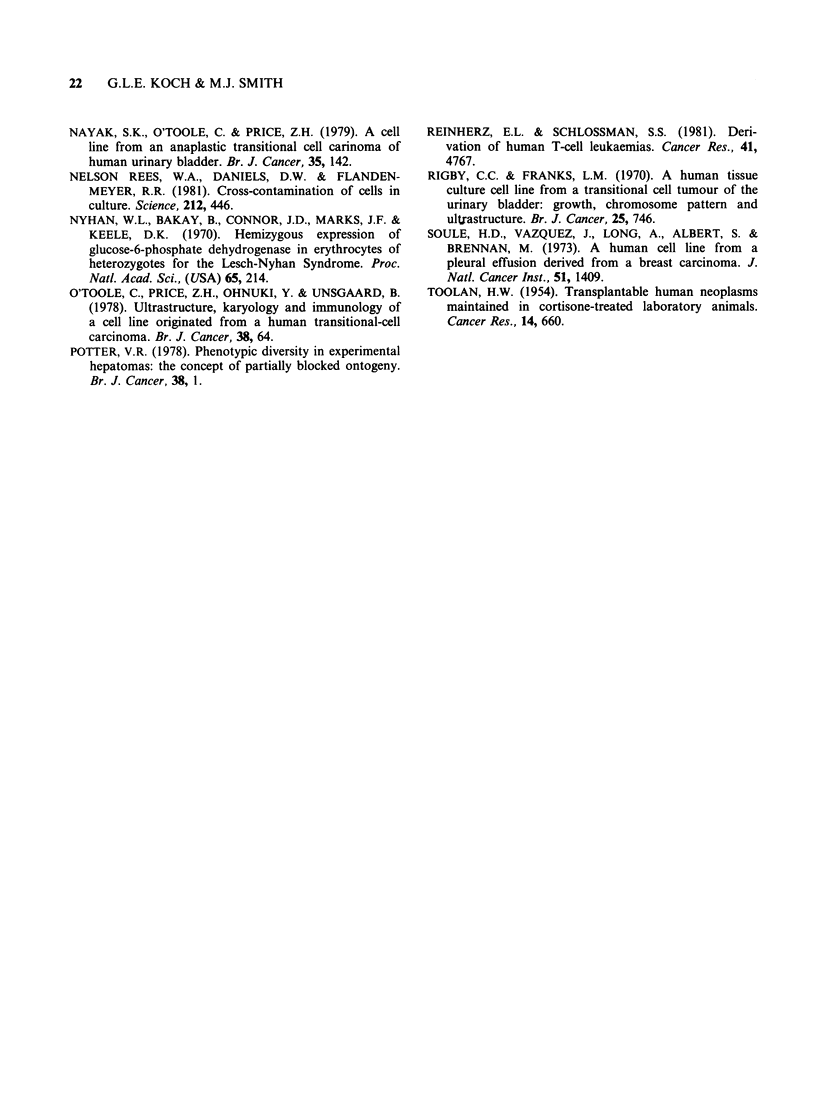# Specificity of antibodies to the purified Con A acceptor glycoproteins of cultured tumour cells.

**DOI:** 10.1038/bjc.1986.3

**Published:** 1986-01

**Authors:** G. L. Koch, M. J. Smith

## Abstract

**Images:**


*Br. J. Cancer* (1986), **53**, 13–22

### Specificity of antibodies to the purified Con A acceptor glycoproteins of cultured tumour cells

G.L.E. Koch & M.J. Smith

*Laboratory of Molecular Biology, Medical Research Council Centre, Hills Road, Cambridge CB2 2QH, UK.*

**Summary** Con A acceptor glycoproteins from the human Molt 4 (T cell leukaemia) and HeLa (endocervical adenocarcinoma) cell lines were purified by affinity chromatography and used for the preparation of rat antisera. Cross-absorption analysis showed that each antiserum contained antibodies which recognised cell surface antigens preferentially expressed by the donor cell line. Molt 4-associated antigens were fully expressed on T cell tumour lines and normal thymocytes, but not on non T cell tumour lines, peripheral blood lymphocytes or other blood cells. Immunofluorescence studies showed that the antigens were preferentially expressed on a sub-population of immature thymocytes. HeLa-associated antigens were only fully expressed on one other epithelial tumour cell in a panel of 17 cell lines. Immunofluorescence studies showed that the HeLa-associated antigens were expressed on normal endocervical adenoepithelium but not on ectocervical, endometrial or intestinal epithelia. Thus purified Con A acceptor glycoproteins of cultured tumour cell lines are potent immunogens for the generation of antibodies recognising lineage-associated differentiation antigens. These antigens should be useful in tumour classification and in the study of normal differentiation.

Two-dimensional gel analysis of the Con A acceptor glycoproteins of cultured tumour cells has shown that the complex glycoprotein repertoires expressed by these cells can be classified into two general groups; constant glycoproteins which are expressed by all tumour cells and normal cells of a species and variable glycoproteins which are only expressed by some types of cells (Koch & Smith, 1982). Since the patterns of variable glycoproteins expressed by cells derived from the same pathway of differentiation are usually similar or even identical (Koch & Smith, 1983) it was concluded that the variable glycoproteins reflect the state of differentiation of the normal precursor cell from which each tumour cell originates. This linkage of the pattern of variable glycoproteins to cell differentiation suggested that they could be of considerable value as markers for the classification of tumours as well as the study of normal differentiation and development. The particular advantages of the Con A acceptor glycoproteins of cultured cells are that each cell type can express up to 50 separate differentiation-linked glycoproteins, they can be readily isolated in a relatively purified form by Con A affinity chromatography and large amounts may therefore be administered to recipient animals for the preparation of polyclonal and/or monoclonal antibodies of the desired specificity.

In this study we have assessed the value of the Con A acceptor glycoproteins of two cultured human tumour cell lines for the generation of antibodies which react with antigens preferentially expressed by the normal cell or tissue from which each tumour was derived. The results show that the anti-glycoprotein antibodies do recognise differentiation antigens associated with the tissues of origin of the respective tumours. Some of these antigens have the novelty of being preferentially associated with the relatively immature cells of a lineage and may therefore provide a systematic approach to the identification and isolation of the early cells of a lineage which appear to be the main source of malignant cells in at least some lineages (Potter, 1978; Greaves, 1981).

Correspondence: G.L.E. Koch.
Received 3 April 1985; and in revised form 16 September 1985.

### Materials and methods

### *Reagents*

RPMI 1640 was purchased as powdered medium from Flow Ltd, reconstituted with glass-distilled water and sterilised by filtration. Penicillin and streptomycin were obtained as sterile solutions from Gibco Ltd. Newborn and foetal calf serum were from Sera-Lab. PBS (2.36 g $Na_2HPO_4$; 1.3 g $NaH_2PO_4 \cdot 2H_2O$; 8 g NaCl pH 7.2 in 1l) and EDTA-saline (8 g NaCl; 1.15 g $Na_2HPO4$; 0.2 g $KH_2PO4$; 0.2 g EDTA pH 7.2 in 1l) were prepared from Analar Chemicals (BDH Ltd) and sterilised by filtration. CNBr-Sepharose and Concanavalin A were from Pharmacia. Lymphoprep™ was from Nyegaard, Nonidet P40 from BDH Ltd and α-methyl mannoside from Sigma. Rartig was purchased from Miles Ltd and iodinated by the Chloramine T method (Hunter & Greenwood, 1962) with Na $^{125}I$ (Amersham 1MS30). Rhodamine and

© The Macmillan Press Ltd., 1986

fluorescein conjugated antibodies (Miles Ltd) were passed through a column of Sephadex G25 fine (Pharmacia) in PBS to remove free fluorochrome and used at a final dilution of 1:20. Rabbit anti transferrin receptor antiserum (Bliel & Bretscher, 1982) was a gift from Dr J.D. Bliel and monoclonal NA 13/4 antibody (McMichael *et al.*, 1979) was a gift from Dr C. Milstein.

*Cells*

HeLa cells were from Dr R.T. Johnson, Department of Zoology, University of Cambridge; Molt 4, JM, HPB, CCRF-CEM, Daudi, HMY and WT52 cells were from Dr C. Milstein, Laboratory of Molecular Biology, Cambridge; HEp-2, NB100, HT29, MCF-7, GCCM and GM1063 cells were from Dr K. Talbot, Ludwig Institute, Cambridge; RT4, J82, T24 and TCC-Sup cells were from Dr C. O'Toole, Department of Pathology, University of Cambridge and LEED cells were from Dr M. Stanley, Department of Pathology, University of Cambridge.

Cell lines were grown in RPMI 1640 medium with 10% newborn or foetal calf serum, 10 ml glutamine, 100 U ml$^{-1}$ penicillin/streptomycin in Corning tissue culture plastic flasks. Suspension cells were harvested by centrifugation at 100 *g* for 5 min. Adherent cells were harvested by incubation at 37°C in EDTA-saline. Human blood cells were isolated from fresh heparinised blood by centrifugation on Lymphoprep. The undiluted blood was layered onto 2 vol of Lymphoprep and centrifuged at 2000 *g* for 20 min at 20°C. The plasma layer was centrifuged at 20,000 *g* for 20 min to obtain a platelet preparation, the interface cells diluted with PBS pelleted at 2000 *g* to obtain a lymphocyte preparation and the red cell pellet washed and used directly. CLL lymphocytes were obtained from a patient using the above procedure.

All cells were washed with PBS before use in absorption and binding tests. Viability was always >90% by the Trypan blue exclusion test.

Thymocytes were obtained from freshly excised thymus from a patient undergoing paediatric cardiac surgery. The cells were teased out into PBS and washed 3 × before use. Cells were stored in 50% foetal calf serum, 10% DMSO in RPMI 1640 medium in liquid nitrogen. Frozen cells were rapidly thawed and fixed immediately in 3% formaldehyde in PBS for 15 min at room temperature to prevent fragmentation.

*Preparation of Con A Sepharose*

CNBr-Sepharose (Pharmacia) was washed and swollen as prescribed by the manufacturers. The swollen resin was coupled to pure concanavalin A (Pharmacia) using 500 mg of Con A per 15 ml of swollen resin. After overnight mixing on rollers the coupled resin was washed, blocked with 1 M ethanolamine-HCl pH 8.0 for 1 h at 4°C, washed and fixed with 0.25% glutaraldehyde in PBS at room temperature for 15 min*. The resin was washed and resuspended in PBS with azide as preservative.

*Purification of Con A acceptor glycoproteins*

Washed cells were suspended in PBS at a concentration of ~10 g cells per 50 ml of suspension. Nonidet P40 was then added to a concentration of 2% and after thorough mixing for ~5 min on ice the dense nuclear material was centrifuged out and the supernatant mixed with 15 ml of Con A Sepharose per 50 ml of cell lysate. This was mixed on rollers for 10–15 h at 4°C and the resin washed 5 × with PBS to remove the excess lysate. The glycoproteins were eluted with 30 ml of 20% α-methyl mannoside in PBS with 2% Nonidet P40 for ~10 h at 4°C. The eluate was dialysed against 10 mM Tris pH 7.4 and concentrated by dialysis against PEG followed by re-dialysis against PBS to remove excess PEG. The sample was adjusted to a final concentration of about $5 \times 10^9$ cell equivalents ml$^{-1}$.

*Immunisation with purified Con A acceptor glycoproteins*

$2 \times 10^9$ cell equivalents (2–4 mg protein for Molt 4 and HeLa glycoproteins) in 600 µl of PBS were mixed with 600 µl of Freunds complete adjuvant and injected intraperitoneally into A0 × Lou strain rats. After 1 month a similar injection was carried out and test bleeds obtained after a further 2 weeks.

*Standard binding assay for antibodies to cells*

Cells ($2 \times 10^5$) were incubated with rat antiserum (total volume 100 µl) in microtite plates for 1 h at 20°C. The cells were washed 3 times and incubated with 50 µl of $^{125}$I-Rartig ($10^6$ c.p.m. ml$^{-1}$) for 1 h at 20°C. After washing, cells were counted in an LKB multi-gamma counter.

For antiserum titrations, antisera were serially diluted starting at a 1:1000 dilution. In the absorption test (see below), absorbed antisera were assayed as above using an antiserum dilution equivalent to 50% of maximal binding to the homologous cell line (i.e. anti-Molt with Molt 4 cells and anti-HeLa with HeLa cells). Pre-bleed sera

*Fixation of Con A was carried out to reduce leaching to the lectin from the resin during elution of glycoproteins to be used for immunisations.

were used as control for non-specific binding. All dilutions and washings were carried out in dilution medium (PBS, 10% foetal calf serum, 0.02% sodium azide).

*Standard absorption assay for antigen expression*

Each cell line was serially diluted starting at $2 \times 10^7$ HeLa cell equivalents (see below) and mixed with anti-serum (anti-Molt 4 or anti-HeLa glycoproteins at 1:50,000 or 1:10,000 dilution respectively) in a final volume of 200 $\mu$l in Luckham LP3 tubes. After incubation on a rolling mixer for 2 h at 4°C, the cells were spun out and the residual antibody activity measured in the standard binding assay. The measure of antigen expression was the maximum absorption (%) achievable by each cell line. Therefore it was necessary to use a starting concentration of cells which was sufficient to ensure a plateau level in the absorption curve for each cell line. Preliminary studies showed that by using a starting cell pellet equivalent in volume to $2 \times 10^7$ HeLa cell equivalents the achievement of the maximum absorption was ensured.

It should be emphasised that because the residual activity represents antibodies directed against antigens completely absent from the test cell line the measure of antigen expression (maximum absorption) for each cell line is independent of cell volume, surface area or antigen density. This assay also permits the comparison of antigen expression between cells and acellular material such as tissue extracts, membranes, etc.

*Preparation of spleen membranes for absorption*

Frozen human spleen was thawed and minced with scissors to yield a coarse suspension in 3 volumes of ice-cold PBS. The suspension was dispersed in a Polytron homogeniser and the dense material removed by centrifugation at 1000 *g*. Membranes were collected by centrifugation at 100,000 *g* for 1 h onto a cushion of 45% sucrose in PBS. The membranes were diluted and pelletted at 100,000 *g* for 1 h to produce a stock suspension. After use in the absorption tests membranes were removed by pelletting at 100,000 *g* for 1 h.

*Immunofluorescence*

Molt 4 and the HeLa glycoprotein antisera (1:100 in dilution medium) were absorbed with human spleen membranes and HT29 colon carcinoma cells respectively to remove antibodies reacting with common antigens. Absorbed Molt 4 glycoprotein antiserum was used at a dilution of 1:200 or 1:1000 and absorbed HeLa glycoprotein antiserum at a dilution of 1:1000. NA13/4 antibody was from a culture supernatant and was used undiluted. Rabbit anti-human transferrin receptor serum was diluted 1:100 in dilution medium. Thymocytes or cryostat sections were fixed with formaldehyde as described by Finan *et al.* (1982) before use. After incubation for 1 h at 20°C with the first antibody, and washing 3 times with dilution medium, samples were incubated with the fluorescent antibody for 1 h at 20°C, washed 3 times with dilution medium, mounted in 50% glycerol in PBS and examined for epifluorescence on a Zeiss microscope. Rat antibodies were developed with Rh-Rartig (1:20) NA13/4 anti-body with Fl-Ramig (1:20) and Rabbit anti-transferrin receptor with Fl-Garig (1:20). For double labelling experiments on thymocytes, the anti-transferrin receptor antibody was used first, blocked with neat rabbit serum for 30 min at 20°C, followed by the rat/anti-rat antibodies. Fluorescent rabbit anti-rat and anti-mouse antibodies showed no cross-reaction and could be used in any sequence. Cryostat sections of human ectocervix, endocervix and endometrium were kindly provided by Dr M. Stanley and sections of human intestinal epithelia were kindly provided by Dr P. Ciclitira. They were stained according to the procedure described by Finan *et al.* (1982).

*Gel electrophoresis*

SDS gel electrophoresis was carried out according to the method of Laemmli (1970) and stained for protein with Coomassie Blue. 2D-gel analysis with $^{125}$I Con A was carried out as described previously (Koch & Smith, 1982).

*Estimation of protein*

Protein was measured by the Coomassie Blue procedure using bovine serum albumin as standard (Bradford, 1976).

### Results

*Isolation of Con A acceptor glycoproteins for immunisation*

Figure 1 shows the composition of purified glycoprotein preparations from the HeLa and Molt 4 cell lines. When SDS gel electrophoresis is used for analysis a few major bands are observed with the conventional protein stain. However, when analysed by 2D gel electrophoresis in conjunction with $^{125}$I Con A overlay it is clear that the preparations contain a large number of Con A binding components. Thus although these preparations are relatively enriched for the Con A acceptor glycoproteins they are still quite complex mixtures of glycoproteins.

**Figure 1** Composition of purified Con A acceptor glycoproteins from cultured tumour cell lines. **(A)** Eluates from Con A Sepharose analysed by SDS gel electrophoresis, and stained for protein with Coomassie Blue. (1) Protein standards (glycogen phosphorylase 95 kD, bovine serum albumin 65 kD, ovalbumin 45 kD, carbonic anhydrase 30 kD). (2) Glycoproteins from Molt 4 cells. (3) Glycoproteins from HeLa cells. **(B)** HeLa glycoproteins analysed by 2D gel and $^{125}$I Con A overlay (Koch & Smith, 1982). **(C)** Molt 4 glycoproteins analysed by 2D gel and $^{125}$I Con A overlay.

**Figure 2** Detection of specific antibodies in antisera to Molt 4 and HeLa glycoproteins. Anti-Molt 4 glycoprotein serum (dil 1:50,000) and anti-HeLa glycoprotein serum (1:10,000) were absorbed with varying amounts of Molt 4 and HeLa cells in the standard absorption assay. Absorbed samples were tested for binding to Molt 4 and HeLa cells respectively. Top. Absorption of anti-Molt 4 by Molt 4 and HeLa cells. Bottom. Absorption of anti-HeLa by Molt 4 and HeLa cells.

### *Antibodies to Molt 4 and HeLa glycoproteins recognise cell surface antigens preferentially expressed by the donor cell line*

Rats immunised with purified glycoproteins from either the Molt 4 or HeLa cell lines produce high levels of antibodies which react with surface antigens from the donor cell line. In some animals the binding titres of these antisera exceed $3 \times 10^5$ showing that the soluble glycoproteins are potent immunogens. Figure 2 shows that whereas Molt 4 cells could completely absorb out the antigens recognised by the anti-Molt 4 glycoprotein antibodies, the HeLa cell could only absorb out ~20% of the activity in the same test system. In the reciprocal experiment using the antibodies to the HeLa cell glycoproteins, Molt 4 cells could only absorb ~20% of the anti-HeLa antibodies.

### *Molt 4 – associated glycoprotein antigens are expressed on T-ALL cell lines and normal thymocytes*

The expression of the antigens recognised by the antibodies to the Molt 4 glycoproteins was examined by absorption analysis with a panel of cell lines and normal cells. In each case the maximum possible absorption was determined by titration (see Figure 2) with adequate amounts of the test sample in the anti-Molt 4/Molt 4 test system. Table I shows that the maximum absorption achieved by the non-T cell lines was ~35% of the total anti-Molt 4 activity. This probably represents the activity towards common antigens in the anti-Molt 4 serum. In contrast, all the T cell tumour lines absorbed the anti-Molt 4 activity completely, indicating that they expressed all the antigens recognised by the serum.

Analysis of normal cells and tissues also indicated that the antigens recognised by the anti-Molt 4 serum were normal differentiation antigens associated with the T cell lineage. Thus, platelets, erythrocytes, peripheral lymphocytes and spleen membranes failed to absorb >50% of the total activity. In contrast the absorption by thymocytes was complete. When the Molt 4 glycoprotein anti-serum was absorbed by spleen membranes, the

**Table I** Expression of Molt 4 glycoprotein antigens by cultured tumour cells, normal cells and tissues.

| *Cell* | *Ref* | *Cell type* | *% Absorption of Molt 4 antibodies* |
|---|---|---|---|
| Molt 4 | a | T-cell leukaemia | 100 |
| JM | a | T-cell leukaemia | 100 |
| HPB | a | T-cell leukaemia | 100 |
| CCRF-CEM | b | T-cell leukaemia | 100 |
| Daudi | c | B-cell leukaemia | 36 |
| Hmy | d | B-cell leukaemia | 25 |
| HeLa | e | Endocervical carcinoma | 15 |
| HEp-2 | f | Laryngeal carcinoma | 15 |
| LEED | g | Cervical squamous carcinoma | 33 |
| RT4 | h | Bladder TC carcinoma | 32 |
| NB100 | i | Neuroblastoma | 34 |
| Erythrocytes | | | 15 |
| Platelets | | | 30 |
| Peripheral blood lymphocytes | | | 37 |
| CLL-lymphocytes | | | 45 |
| Spleen membranes | | | 35 |
| Thymocytes | | | 100 |

Anti-Molt 4 glycoprotein antibodies were completely absorbed by each test cell as described in **Materials and methods** and residual activity tested with Molt 4 cells in the standard binding assay.

[a]Minowada (1980); [b]Foley *et al.* (1965); [c]Klein *et al.* (1968); [d]Edwards *et al.* (unpublished); [e]Gey *et al.* (1952); [f]Toolan (1954); [g]Not available; [h]Rigby & Franks (1970); [i]Not available.

residual antibodies bound strongly to thymocytes but weakly to peripheral blood lymphocytes (Figure 3).

Studies with monoclonal antibodies have shown that human thymocytes express a major antigen HTA (McMichael *et al.,* 1979) which is not expressed on mature T cells. Several observations showed that the thymocyte antigens recognised by the anti-Molt 4 serum were not HTA. Double immunofluorescence studies on normal thymocytes with anti-HTA and anti-Molt 4 antibodies reveal clear differences between their pattern of expression (Figure 4). First, the HTA and Molt 4 antigens are not expressed on the same sub-population of cells. In fact, cells which are high in HTA are usually low in the Molt 4 antigens and vice versa (Figure 4C, D). When the two sets of antigens are expressed by the same cell the pattern of expression is also different. The HTA usually gives a regular punctuate pattern over the whole cell surface whereas the pattern of the Molt 4 antigens is more uniform and distinct from the HTA pattern. Thus it appears that the Molt 4 antigens are distinct from the human thymocyte antigen.

Evidence was also obtained that some of the Molt 4 antigens are preferentially expressed on the

**Figure 3** Specificity of anti-Molt 4 glycoprotein antibodies after absorption with spleen membranes. Crude human spleen membranes were prepared as described in **Materials and methods**, titrated for maximum absorption and the fully absorbed serum tested for binding to human thymocytes (THY) and peripheral blood lymphocytes (PBL) in the standard binding assay. Thymocytes and lymphocytes were fixed with formaldehyde as described in **Materials and methods**.

**Figure 4** Immunofluorescence labelling of human thymocytes with anti-Molt 4 glycoprotein antibody. Anti-Molt 4 glycoprotein serum (dil 1:100) was absorbed once with spleen membranes and used for immunofluorescence with fixed thymocytes as described in **Materials and methods**. **(A)** Cells labelled with absorbed antibody at 1:200 final dilution. **(B)** Cells labelled with absorbed antibody at 1:1000 final dilution. **(C, D)** Cells labelled with NA134 (anti-HTA monoclonal antibody) + Fl-Ramig followed by absorbed anti-Molt 4 glycoprotein antibody (1:200) + Rh-Rartig. Arrows show the same cells in the two fields **(C)** Fl-label; **(D)** Rh-label. **(E, F)** Cells labelled with rabbit anti-transferrin receptor antibody + FITC-Garig, blocked with normal rabbit serum followed by absorbed anti-Molt 4 glycoprotein serum (1:1000) + Rh-Rartig. **(E)** FITC label; **(F)** Rh label.

very immature thymic blast cells. When thymocytes were stained with relatively high dilutions of antibody the general staining was decreased but a sub-population of cells still showed strong staining (Figure 4B). These cells usually had an irregular outline and were amongst the largest cells in the thymocyte preparations. Double immunofluorescence with anti-transferrin receptor antibody and anti-Molt 4 antibody showed that these were also the cells which expressed the highest levels of the transferrin receptor (Figure 4E, F). Since high expression of the transferrin receptor is associated with the immature thymic blast cells (Greaves, 1981; Reinharz & Schlossmann, 1981) these observations suggest that the Molt 4 antigens are also preferentially expressed by the immature thymic blasts.

*Antibodies to HeLa glycoproteins recognise antigens expressed preferentially by HeLa cells and normal endocervical epithelium*

The antibodies to the HeLa glycoproteins were examined by absorption analysis with a panel of cultured tumour cell lines (Table II). Absorption by lymphoblastoid cell lines did not exceed ~40%. Other cell lines from various epithelial tissues showed somewhat higher levels of absorption i.e. up to 75%. When the HeLa glycoprotein antiserum was completely absorbed with one cell line (HT29) and then re-absorbed with four other cell lines (Molt 4, LEED, RT4 and NB100) no additional absorption occurred, indicating that absorption is not generally additive. Only one cell line examined so far was able to absorb out all the anti-HeLa activity i.e. the laryngeal carcinoma line Hep2. These studies indicated that a significant proportion of the antibodies to HeLa glycoproteins recognised antigens preferentially expressed on the HeLa cell line.

In order to determine whether the HeLa-associated antigens were expressed by cells of the endocervical epithelium, the putative tissue of origin of the HeLa cell (Jones *et al.*, 1970) cryostat sections were examined by immunofluorescence staining with the anti-HeLa serum after absorption with the HT29 cell line to remove antibodies to common epithelial antigens. Figure 5 shows sections through the endocervical glands which exhibit a characteristic punctate pattern of staining associated with the outer membranes of epithelial cells.

The specificity of the staining for the endocervical epithelium was examined by immunofluorescence with cryostat sections from ectocervical, endometrial and intestinal epithelia. In all cases the staining was not significant. When the preimmune serum or antiserum absorbed with HeLa cells was used the staining of the endocervical cells was abolished (unpublished observations). Thus the HeLa-associated antigens are preferentially expressed by the cells of the endocervical epithelium.

**Table II** Expression of HeLa glycoprotein antigens by cultured tumour cells.

| *Cell* | *Ref* | *Type* | *Maximum absorption* (%) *of anti HeLa GP serum* |
|---|---|---|---|
| HeLa | a | Adenocarcinoma, cervix | 100 |
| LEED | b | Squamous carcinoma, cervix | 16 |
| HT29 | c | Adenocarcinoma, colon | 45 |
| HEP2 | d | Carcinoma, larynx | 100 |
| MCF7 | e | Carcinoma, breast | 16 |
| RT4 | f | Transitional cell carcinoma, bladder | 15 |
| J82 | g | Transitional cell carcinoma, bladder | 68 |
| T24 | h | Transitional cell carcinoma, bladder | 55 |
| TCC Sup | i | Transitional cell carcinoma, bladder | 75 |
| Molt 4 | j | T cell leukaemia | 25 |
| Hmy | k | B cell leukaemia | 12 |
| Daudi | l | Burkitts lymphoma | 12 |
| WT52 | m | B cell leukaemia | 42 |
| GCCM | n | Glioma | 63 |
| GM1063 | o | Fibroblast | 55 |
| Willis | p | Fibroblast | 66 |
| NB100 | q | Neuroblastoma | 30 |

Anti-HeLa glycoprotein antibodies were completely absorbed by each test material as described in **Materials and methods** and residual activity tested with HeLa cells in the standard binding assay.

[a]Gey *et al.* (1952); [b]Not available; [c]Fogh *et al.* (1977); [d]Toulan (1954); [e]Soule *et al.* (1973); [f]Rigby & Franks (1970); [g]O'Toole *et al.* (1978); [h]Bubenik *et al.* (1970); [i]Nayak *et al.* (1979); [j]Minowada (1980); [k]Edwards *et al.* (unpublished); [l]Klein *et al.* (1968); [m]Not available; [n]Garson *et al.* (1981); [o]Nyhan *et al.* (1970); [p]Not available; [q]Not available.

### Discussion

These studies show that purified Con A acceptor glycoproteins of cultured tumour cell lines are potent immunogens for the production of antibodies with a substantial specificity towards the immunising cell line. A major contributing factor to this is the use of large amounts of purified glycoproteins from cultured cell lines as immunogens. The need to use up to 10 g of tumour cell equivalents in such immunisations was suggested by the preliminary studies which showed that $\mu$g quantities of a soluble antigen like chicken ovalbumin were required to generate a satisfactory antibody, and that the 'specific' glycoproteins expressed by the cultured tumour lines are very minor components of such cells. This illustrates one of the advantages of using suitably characterised molecules as immunogens since it can reduce at least some of the uncertainty involved in all such immunisations. It also emphasises the value of using purified immunogens since adequate amounts of minor components can be administered during immunisation.

The main object of these studies was to examine the specificity of the antibodies obtained when purified Con A acceptor glycoproteins were used as immunogens. It is clear that such antibodies show significant specificity towards antigens expressed by the donor cell line. Thus in the Molt 4/HeLa cross-comparison ~80% of the antibodies which react with the donor cell line fail to react with the other line (Figure 1). In the more general comparisons with unrelated cultured tumour cell lines the non-specific reactivity is usually around ~50% (Tables I and II). Thus it is possible to produce an antiserum of high titre and specificity towards the donor cell line by a single absorption with an unrelated line.

The antigens which are preferentially expressed by the Molt 4 cell line are also fully expressed on other cell lines of the T cell lineage. The implication of this linkage to tumour cells derived from a specific lineage is that the antigens are differentiation markers associated with T cell development. This was directly confirmed by the observation that the antigens are fully expressed on normal human

**Figure 5** Expression of HeLa-associated glycoprotein antigens on human endocervical epithelium. **(A)** Anti-HeLa glycoprotein antibody (1:100 dil) was absorbed once with HT29 cells to completely remove antibodies reacting with common antigens. Cryostat sections were stained as described by Finan *et al.* (1982) using Rh-Rartig as second layer. Sections through normal endocervical glandular ducts are shown. Control (pre-immune and HeLa-absorbed) sera showed no staining of parallel sections.

thymocytes, the immature precursors of T cells. Similarly, the HeLa-associated antigens were found on normal endocervical epithelium, the putative tissue of origin of the HeLa cell but not on the other epithelial tissues examined. The main exceptions to this specificity for cells of the same lineage as the donor cell is the HEp-2 cell line which is derived from laryngeal epithelium (Toolan, 1954) but expressed all the antigens recognised by the anti-HeLa antibodies. This similarity in the antigen profiles of the HEp-2 and HeLa cell lines could reflect the properties of the normal epithelial cells from which they originated. However, it has been shown that HEp-2 cells from a number of sources (including the ATCC collection) express the specific markers such as the marker chromosomes, G6PD type A isozyme and lack of Y chromosome associated with the HeLa cell line (Nelson-Rees *et al.,* 1981). Thus the similarity in the antigen profiles of HEp-2 and HeLa may reflect this putative contamination rather than the properties of authentic laryngeal carcinoma cells. It is worth noting that in view of the numerous reports of HeLa contamination of cell lines, the simple absorption test using the anti-HeLa glycoprotein serum used in these studies could provide a rapid screening assay for HeLa cell contamination in laboratory cultures.

It is interesting that there is no evidence in these studies for antigens which are not expressed by the normal cells from which the tumour cells arise. Thus thymocytes fully express all the antigens detected by the anti-Molt 4 antibodies. It is remarkable that the HeLa cell line which has been in cell culture for several decades (Gey *et al.,* 1952) under poorly controlled conditions should retain the glycoprotein antigen profile of the normal tissue from which it was derived. However these observations are consistent with previous studies showing that the glycoprotein fingerprints of cultured tumour cells do not change during long-term tissue culture (Koch *et al.,* 1983). This suggests a general approach to the classification of tumour cell lines where there is an uncertainty about their origin i.e.: anti-glycoprotein antibodies can be prepared, absorbed till they are specific towards the donor cell line, and then used to examine antigen expression on prospective normal tissues.

The absence of most of the Molt 4-associated antigens on mature T cells shows that they are preferentially expressed on the relatively immature cells of the T cell lineage. This is not surprising since it is well-established that T-ALL is associated with the proliferation of cells in the thymus (Reinherz & Schlossman, 1981). However the indications that the Molt 4-associated antigens are preferentially expressed by the very immature thymic-blast cells is more interesting since there are no known markers which are both T lineage specific and preferentially expressed by the cells at the earliest stages of commitment to this lineage. Such markers could be particularly valuable in the isolation and characterisation of pure populations of prothymocytes.

Although purified preparations enriched for the specific glycoproteins were used as immunogens, it has not yet been directly demonstrated that these are the antigens actually recognised by the specific antibodies. The immunochemical analysis required for such studies were precluded by the limited amounts of rat anti-serum available. The use of rats rather than larger species such as rabbit or sheep was dictated by the intention to go on to the production of monoclonal antibodies if the anti-glycoprotein sera proved promising. It also remains to be determined to what extent the protein and carbohydrate moieties of the glycoprotein immunogens contribute to the specificity of the antibodies. Studies with monoclonal antibodies to tumour cells surfaces have led to the suggestion that the differentiation antigens expressed on cell surfaces are predominantly carbohydrate moieties (Feizi, 1985). However it has not been excluded that this selectivity for carbohydrate antigens is a reflection of the approaches used in the production of monoclonal antibodies (Feizi, 1985) and not necessarily a reflection of the absence of protein antigens generally. A detailed analysis of the biochemical nature of the antigens recognised by the specific antibodies in the anti-glycoprotein sera could be instructive.

The main outcome of these studies is that they suggest a general approach to the identification of lineage-associated differentiation antigens. It relies on the use of a pure cultured tumour cell population as the source of a purified set of immunogens which can be shown by independent biochemical analyses to be expressed in a lineage-associated fashion. It is expected that by combining these well defined immunogens with the hybridoma technique (Köhler & Milstein, 1975) novel markers for tumour classification and cell differentiation can be identified in a reliable and predictable approach.

### References

BLEIL, J.D. & BRETSCHER, M.S. (1982). Transferrin receptor and its recycling in HeLa cells. *EMBO J.*, **1**, 351.

BRADFORD, M.M. (1976). A rapid and sensitive method for the quantitation of microgram quantities of protein utilising the principle of protein-dye binding. *Anal. Biochem.*, **72**, 248.

BUBENIK, J., PERLMANN, P., HELMSTEIN, K. & MORBERGER, G. (1970). Cellular and humoral responses to human urinary bladder carcinomas. *Int. J. Cancer*, **5**, 310.

FEIZI, T. (1985). Demonstration by monoclonal antibodies that carbohydrate structures of glycoproteins and glycolipids are onco-developmental antigens. *Nature*, **317**, 53.

FINAN, P.J., GRANT, R.M., DE MATTOS, C. & 4 others. (1982). Immunohistochemical techniques in the early screening of monoclonal antibodies to human colonic epithelium. *Br. J. Cancer*, **46**, 9.

FOGH, J., FOGH, J.M. & ORFEO, T. (1977). One hundred and thirty seven cultured human tumour cell lines producing tumours in nude mice. *J. Natl. Cancer Inst.*, **59**, 221.

FOLEY, G.E., LAZARUS, H., FARBER, S., UZMAN, B.G., BOONE, B.A. & McCARTHY, R.E. (1965). Continuous culture of human lymphblasts from peripheral blood of a child with acute leukaemia. *Cancer*, **18**, 522.

GARSON, G.A., QUINDLEN, E.A. & KORNBLITH, P.L. (1981). Complement fixation of IgM and IgC auto-antibodies on cultured huam glial cells. *J. Neurosurg.*, **55**, 19.

GEY, G.O., COFFMAN, W.D. & KUBICEK, M.T. (1952). Tissue culture studies of the proliferative capacity of cervical carcinoma and normal epithelium. *Cancer Res.*, **12**, 264.

GREAVES, M.F. (1981). Analysis of the clinical and biological significance of lymphoid phenotypes in acute leukaemia. *Cancer Res.*, **41**, 4752.

HUNTER, W.M. & GREENWOOD, P.C. (1962). Preparation of Iodine-131 labelled human growth hormone of high specific activity. *Nature*, **194**, 495.

JONES, H.W., McKUSICK, V.A., HARPER, P.S. & WUU, K.D. (1971). The HeLa cell and a reappraisal of its origin. *Obstet. Gynecol.*, **38**, 945.

KLEIN, E., KLEIN, G., NADKARNI, J.S., NADKARNI, J.J., WIGZELL, H. & CLIFFORD, P. (1968). Surface IgM-kappa specificity on a Burkitt's lymphoma cell *in vitro* and in derived cell lines. *Cancer Res.*, **28**, 1300.

KOCH, G.L.E. & SMITH, M.J. (1982). Analysis of the glycoproteins of murine tumour cell lines with $^{125}I$ Concanavalin A in two-dimensional electrophoresis gels. *Eur. J. Biochem.*, **128**, 107.

KOCH, G.L.E. & SMITH, M.J. (1983). Con A acceptor glycoproteins: A new type of marker for the classification of tumour cells. *Br. J. Cancer*, **47**, 527.

KOCH, G., SMITH, M.J., GRANT, A.G. & HERMAN-TAYLOR, J. (1983). Stability of the glycoproteins from a primary human pancreatic carcinoma during cell culture and *in vivo* passage in nude mice. *Br. J. Cancer*, **47**, 537.

KOHLER, G. & MILSTEIN, C. (1975). Continuous cultures of fused cells secreting antibody of pre-defined specificity. *Nature*, **256**, 495.

LAEMMLI, U.K. (1970). Cleavage of structural proteins during the assembly of the head of bacteriophage T4. *Nature*, **227**, 680.

McMICHAEL, A.J., PILCH, J.R., GALFRE, G., MASON, D.Y., FABRE, J.W. & MILSTEIN, C. (1979). A human thymocyte antigen defined by a hybrid myeloma monoclonal antibody. *Eur. J. Immunol.*, **9**, 205.

MINOWADA, J. (1980). Marker profiles of human leukaemia and lymphoma cell lines. *J. Cancer Res. Clin. Oncol.*, **101**, 91.

MINOWADA, J., MINATO, K., SRIVASTAWA, B.I.S. & 8 others. (1982). A model scheme of human hematopoietic cell differentiation as determined by leukemia-lymphoma study T-cell lineages. In *Current Concepts in Human Immunology and Cancer Immunomodulation*, Serrou *et al.* (eds) p. 75. Elsevier: Amsterdam.

NAYAK, S.K., O'TOOLE, C. & PRICE, Z.H. (1979). A cell line from an anaplastic transitional cell carinoma of human urinary bladder. *Br. J. Cancer*, **35**, 142.

NELSON REES, W.A., DANIELS, D.W. & FLANDENMEYER, R.R. (1981). Cross-contamination of cells in culture. *Science*, **212**, 446.

NYHAN, W.L., BAKAY, B., CONNOR, J.D., MARKS, J.F. & KEELE, D.K. (1970). Hemizygous expression of glucose-6-phosphate dehydrogenase in erythrocytes of heterozygotes for the Lesch-Nyhan Syndrome. *Proc. Natl. Acad. Sci.*, (*U*SA) **65**, 214.

O'TOOLE, C., PRICE, Z.H., OHNUKI, Y. & UNSGAARD, B. (1978). Ultrastructure, karyology and immunology of a cell line originated from a human transitional-cell carcinoma. *Br. J. Cancer*, **38**, 64.

POTTER, V.R. (1978). Phenotypic diversity in experimental hepatomas: the concept of partially blocked ontogeny. *Br. J. Cancer*, **38**, 1.

REINHERZ, E.L. & SCHLOSSMAN, S.S. (1981). Derivation of human T-cell leukaemias. *Cancer Res.*, **41**, 4767.

RIGBY, C.C. & FRANKS, L.M. (1970). A human tissue culture cell line from a transitional cell tumour of the urinary bladder: growth, chromosome pattern and ultrastructure. *Br. J. Cancer*, **25**, 746.

SOULE, H.D., VAZQUEZ, J., LONG, A., ALBERT, S. & BRENNAN, M. (1973). A human cell line from a pleural effusion derived from a breast carcinoma. *J. Natl. Cancer Inst.*, **51**, 1409.

TOOLAN, H.W. (1954). Transplantable human neoplasms maintained in cortisone-treated laboratory animals. *Cancer Res.*, **14**, 660.